# Acute responsiveness to single leg cycling in adults with obesity

**DOI:** 10.14814/phy2.15539

**Published:** 2022-12-21

**Authors:** Kevin J. Gries, Corey R. Hart, Hawley E. Kunz, Zachary Ryan, Xiaoyan Zhang, Mojtaba Parvizi, Yuanhang Liu, Surendra Dasari, Ian R. Lanza

**Affiliations:** ^1^ Endocrine Research Unit, Division of Endocrinology Department of Internal Medicine, Mayo Clinic Rochester Minnesota USA; ^2^ Department of Physical Therapy, School of Health Professions Concordia University of Wisconsin Mequon Wisconsin USA; ^3^ Air Force Research Laboratory, 711th Human Performance Wing, Wright Patterson Air Force Base Dayton Ohio USA; ^4^ Department of Geriatrics Shanghai Jiaotong University Affiliated Sixth People's Hospital Shanghai China; ^5^ Department of Biomedical Statistics and Informatics, Mayo Clinic Rochester Minnesota USA

**Keywords:** amino acid metabolism, exercise, protein synthesis, transcriptome

## Abstract

Obesity is associated with several skeletal muscle impairments which can be improved through an aerobic exercise prescription. The possibility that exercise responsiveness is diminished in people with obesity has been suggested but not well‐studied. The purpose of this study was to investigate how obesity influences acute exercise responsiveness in skeletal muscle and circulating amino metabolites. Non‐obese (NO; *n* = 19; 10F/9M; BMI = 25.1 ± 2.8 kg/m^2^) and Obese (O; *n* = 21; 14F/7M; BMI = 37.3 ± 4.6 kg/m^2^) adults performed 30 min of single‐leg cycling at 70% of VO_2_peak. ^13^C_6_‐Phenylalanine was administered intravenously for muscle protein synthesis measurements. Serial muscle biopsies (vastus lateralis) were collected before exercise and 3.5‐ and 6.5‐h post‐exercise to measure protein synthesis and gene expression. Targeted plasma metabolomics was used to quantitate amino metabolites before and 30 and 90 min after exercise. The exercise‐induced fold change in mixed muscle protein synthesis trended (*p* = 0.058) higher in NO (1.28 ± 0.54‐fold) compared to O (0.95 ± 0.42‐fold) and was inversely related to BMI (R^2^ = 0.140, *p* = 0.027). RNA sequencing revealed 331 and 280 genes that were differentially expressed after exercise in NO and O, respectively. Gene set enrichment analysis showed O had six blunted pathways related to metabolism, cell to cell communication, and protein turnover after exercise. The circulating amine response further highlighted dysregulations related to protein synthesis and metabolism in adults with obesity at the basal state and in response to the exercise bout. Collectively, these data highlight several unique pathways in individuals with obesity that resulted in a modestly blunted exercise response.

## Graphical abstract

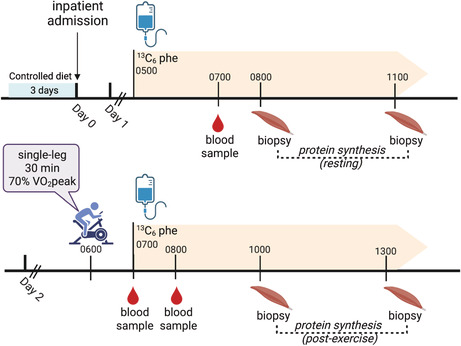

## 1 INTRODUCTION

The global obesity trend and its societal impact necessitate continued efforts to identify new ways to lessen the burden of obesity. Toward this goal, skeletal muscle is a tissue of interest. Skeletal muscle from individuals with obesity exhibits mitochondrial impairments (Ritov et al., 2005), oxidative stress (Anderson et al., 2009), ectopic lipid accumulation (Kelley & Goodpaster, 2001), and insulin resistance (DeFronzo & Tripathy, 2009) that are believed to contribute to obesity‐related comorbidities (Blüher, 2019). These maladaptive features, along with the key role of skeletal muscle in macronutrient metabolism, have motivated initiatives to target skeletal muscle health as a strategy to improve health in people with obesity. Physical activity is recognized as a frontline strategy to enhance skeletal muscle mitochondrial function (Holloszy, 1967), upregulate antioxidant defenses (Gomes et al., 2020), suppress ectopic lipid accumulation (Tarnopolsky et al., 2007), and enhance insulin sensitivity (Richter et al., 1982), overall metabolic health and physical function (Hansen et al., 2009).

The benefits of physical activity are clear, but there is considerable interindividual heterogeneity in the response to exercise. For example, recent analysis of 8 different exercise training studies demonstrates a wide range of individual responses in cardiorespiratory fitness (Ross et al., 2019). These studies spark debate over the concept of exercise non‐responders and how to define classes of responders. Fewer studies have interrogated the impact of obesity on exercise responsiveness from the standpoint of molecular and cellular events in skeletal muscle. The ability to upregulate muscle protein synthesis (i.e., anabolic response) underlies many favorable adaptations to exercise such as muscle mass and mitochondrial biogenesis. While controversy remains, data generally suggest that individuals with obesity exhibit attenuated anabolic responses to nutritional stimuli (Beals et al., 2016, 2019; Guillet et al., 2009; Kouw et al., 2019; Paulussen et al., 2021; Smeuninx et al., 2017). However, even more ambiguous, are the effects of obesity on anabolic response to exercise. There is some evidence of blunted anabolic response to a single bout of resistance exercise in people with obesity based on measurements of myofibrillar protein synthesis and anabolic signaling pathways in skeletal muscle (Beals et al., 2018). Another study demonstrated attenuated expression and phosphorylation of key regulators of mitochondrial biogenesis in obese compared to lean skeletal muscle following a single bout of exercise (De Filippis et al., 2008). In contrast, others suggest that the anabolic response to resistance exercise is unimpaired in young, active adults with obesity (Hulston et al., 2018), emphasizing the need for additional painstakingly controlled studies to help resolve this question. Building on our recent data linking metabolic health with inflammatory signals in individuals with obesity (Kunz et al., 2021), as well as our preliminary work mapping the exercise responsive transcriptome, proteome, and phosphoproteome in people with and without obesity (Vanderboom et al., 2022), we sought to further characterize acute exercise response in individuals across a wide BMI range. The current study demonstrates that the increment in muscle protein synthesis after a single bout of exercise was diminished with increasing BMI. A combination of targeted metabolomics and whole muscle transcriptome analysis revealed pathways involved in cell‐to‐cell communication, energy metabolism, and protein turnover were uniquely influenced by a single exercise bout in people with and without obesity. Together, these data demonstrate that the anabolic response to acute exercise is modestly diminished with increasing BMI and accompanied by distinct obesity‐related and exercise responsive molecular patterns in circulation and in skeletal muscle tissue.

## 2 METHODS

### 2.1 Study participants

Forty (24 females, 16 males) adults aged 30–55 years with BMI ranging from 19–46 kg/m^2^ were recruited from the local Rochester, MN community to participate in the study (Table 1). One participant (non‐obese) did not complete the study and dropped out prior to the acute exercise testing session and was only included in baseline data. Participants were weight‐stable (self‐reported; weight maintained ±2.5 kg in the previous 6 months) and did not participate in structured exercise training (self‐reported activity levels <30 min of exercise 3 times per week). Potential participants' medical histories were reviewed, and participants were excluded if they had anemia, diagnosed diabetes, coronary artery or macrovascular disease, kidney disease, untreated thyroid disease, blood clotting disorders, or any disease or condition that would preclude participation in any study procedure or increase the risks of the study. Exclusion criteria included: fasting plasma glucose >125 mg/dl at time of screening, pregnant or breastfeeding females, tobacco use, consumption >4 oz alcohol per day, other substance abuse disorders, or taking medications that may affect the outcomes of the study or increase the risk of study procedures (e.g., warfarin group medications, metformin, tricyclic antidepressants, benzodiazepines, opiates, barbiturates, anticoagulants). This exercise study was approved by the Mayo Clinic Institutional Review Board (#16–000437), registered at https://clinicaltrials.gov (#NCT02732509) and conducted in accordance with the Declaration of Helsinki. All participants provided informed written consent.

**TABLE 1:** Subject characteristics

	Non‐obese (NO)	Obese (O)	*p* value	Correlation with BMI
*N* (male/female)	19 (9/10)	21 (7/14)	—	—
BMI (kg/m^2^)	25.1 ± 2.8*	37.3 ± 4.7	<0.001	—
Age (years)	37 ± 6	41 ± 8	0.134	*r* = 0.244 *p* = 0.130
Height (cm)	170.0 ± 9.0	169.3 ± 8.2	0.796	*r* = −0.039 *p* = 0.811
Mass (kg)	73.0 ± 12.7*	106.9 ± 16.0	<0.001	*r* = 0.901 *p* < 0.001
Body fat (%)	31.2 ± 6.3*	47.8 ± 6.5	<0.001	*r* = 0.860 *p* < 0.001
Circulating inflammatory profile				
WBC (x10^9^/ml)	6.12 ± 1.43	6.44 ± 1.49	0.500	*r* = 0.174 *p* = 0.290
ESR (mm/h)	6.21 ± 9.47	9.20 ± 9.99	0.263	*r* = 0.402 *p* = 0.011
CRP (mg/dl)	0.139 ± 0.158*	0.562 ± 0.891	0.044	*r* = 0.455 *p* = 0.003
IL‐6 (pg/ml)	3.81 ± 2.95	5.77 ± 4.32	0.100	*r* = 0.106 *p* = 0.515
TNFa (pg/ml)	0.783 ± 0.266*	0.971 ± 0.203	0.018	*r* = 0.456 *p* = 0.003

*Note*: Data presented are mean ± SD. More detailed characteristics including body composition, blood chemistry, inflammatory markers, adipose tissue characteristics, and mitochondrial function can be found in Kunz et al., 2021 (Kunz et al., 2021).

Abbreviations: CRP, C‐reactive protein; ESR, Erythrocyte sedimentation rate; TNFa, Tumer necrosis factor alpha; WBC, White blood cell count.

* *p* < 0.05 versus O.

### 2.2 Outpatient testing

Following consent and screening, participants underwent body composition measurements by dual‐energy x‐ray absorptiometry (DEXA, Lunar iDXA, GE Healthcare). Following the DEXA, subjects performed a whole‐body oxygen consumption test (VO_2_peak) on a single‐leg recumbent ergometer. The ergometer was equipped with a weighted pedal on the right side to provide a counterweight during cycling. The initial workload was set to 25 watts for 2 min, followed by a stepped increase in workload of 10 watt increments every 60 s. Breath‐by‐breath indirect calorimetry (Ultima CPX, Medical Graphics) was used for gas exchange measurements to determine VO_2_peak. Heart rhythm and rate and oxygen saturation were measured by 12‐lead electrocardiogram and pulse oximetry, respectively, and blood pressure and rating of perceived exertion (RPE, Borg Scale) were measured at 2‐min intervals throughout the test. The test was terminated when participants were unable to maintain a cadence of at least 60 revolutions per minute despite strong verbal encouragement. VO_2_ peak was identified as the highest average VO_2_ over a 15 s interval.

### 2.3 Inpatient study

**FIGURE 1:**
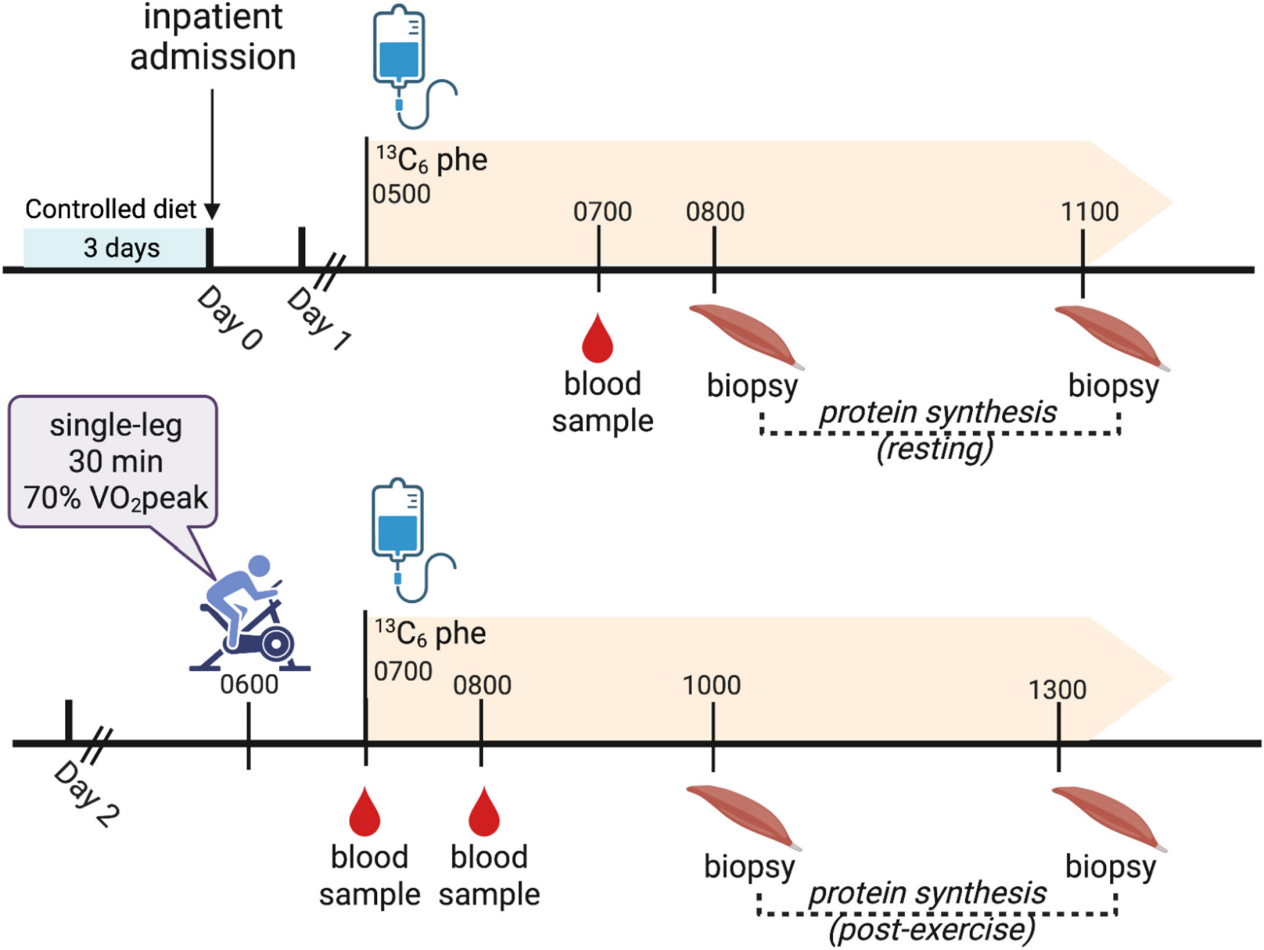
Timeline of the inpatient study period. To assess exercise responsiveness, participants were admitted to the Mayo Clinic clinical research and trials unit for approximately 48 h. Briefly, Day one consisted of continuous infusion of a ^13^C6‐phenylalanine tracer beginning at 0500 h during which percutaneous muscle biopsies of the *vastus lateralis* were performed at 0800 h and 1100 h to assess basal gene expression and fractional synthesis rate. Subjects remained rested the remainder of the day. At 0600 h the following morning, participants performed 30 min of single leg cycling at 70% of the peak power that was achieved on the graded exercise test. After exercise, the continuous ^13^C_6_‐phenylalanine tracer infusion was re‐initiated and muscle biopsies were performed on the *vastus lateralis* muscle of the exercised leg 3.5‐ and 6.5‐h following completion of the exercise bout to measure exercise‐induced gene expression and exercise‐induced fractional synthesis rate. Blood samples were taken at 0700 on Day one, and 30‐ and 90‐min post‐exercise on Day two and analyzed for circulating amine and acyl metabolites.

Participants were admitted to the Mayo Clinic Clinical Research and Trials Unit (CRTU) for approximately 48 h. For 3 days prior to admission, participants were provided with all meals prepared by the dietetics staff of the Metabolic Research Kitchen to achieve weight‐maintenance with standardized macronutrient composition (20% protein, 50% carbohydrate, 30% fat). Participants reported to the Metabolic Kitchen each morning for body weight measurements and breakfast. Lunch and dinner were provided as prepared meals to‐go. On the evening of the third day of standardized meals, participants reported to the inpatient CRTU at approximately 1700 h. An evening meal was provided at 1800 h, and participants remained fasting overnight until completion of the muscle biopsies the following day. The following morning, resting muscle protein synthesis rates were measured in the right leg through intravenous infusion of isotopically labeled ^13^C_6_‐Phenylalanine, serial muscle biopsies, and mass spectrometry to measure the incorporation of amino acid isotope into muscle proteins (Lalia et al., 2017). At 0500 h, ^13^C_6_‐Phenylalanine was administered intravenously through a primed (1.5 mg·kg FFM^−1^), continuous infusion (1.5 mg·kg FFM^−1^·hr^−1^), followed by percutaneous biopsies of the right *vastus lateralis* at 0800 h and 1100 h under local anesthesia (2% lidocaine) using a modified Bergstrom needle. Muscle tissue was rapidly blotted with sterile gauze, weighed at the bedside, frozen in liquid nitrogen, and transferred to −80°C freezer for storage. Participants were provided with weight maintaining afternoon and evening meals and then remained fasting overnight. At 0600 h the following morning, participants performed an acute bout of exercise using the left leg. Participants performed 30 min of single‐leg cycling at a watt load corresponding to 70% of single‐leg VO_2_peak. Watt load was lowered if needed and was recorded. To account for any changes in watt load during the exercise bout, exercise workload (kJ) was calculated as [power (watts) × minutes]. Muscle biopsies were performed on *vastus lateralis* muscle of the exercised leg 3.5‐ and 6.5‐h following completion of the exercise bout and processed as described above. Blood samples for metabolomics were taken at 0700 on Day 1 (basal), and 30‐ and 90‐min post‐exercise. The two‐day inpatient study period is visually shown in Figure 1.

### 2.4 Muscle protein synthesis

Isotopic enrichment in muscle tissue fluid and muscle protein pools were measured by HPLC and tandem mass spectrometry (MS/MS) as previously described (Lanza et al., 2012, 2013; Robinson et al., 2014; Zabielski et al., 2013). Briefly, total mixed muscle protein (MMP) as well as tissue fluid free amino acid fractions were isolated from ~10 mg of muscle sample. Tissue fluid was extracted from ~20 mg of pulverized tissue with 5% sulfosalicylic acid. The remaining protein was hydrolyzed overnight in 6 N HCl at 110°C. Hydrolyzed protein and TF samples were purified using cation exchange columns (AG 50 W‐X8 resin; Bio‐Rad), dried, and derivatized to isobutyl esters. Samples were analyzed by HPLC and tandem mass spectrometry (MS/MS). Data acquisition was performed in positive electrospray ionization mode selecting ion monitoring at 222.4 > 121.6 and 226.4 > 125.6 for the m + 2 and m + 6 fragments of phenylalanine and [^13^C_6_]phenylalanine, respectively. The molar percent excess was calculated against a 6‐point enrichment standard curve. Fractional synthesis rates (FSR) of the mixed muscle protein were calculated from the change in [^13^C_6_]phenylalanine enrichment over the two serial muscle biopsies (E_2_−E_1_) and the average IE of [^13^C_6_]phenylalanine in the tissue fluid as the precursor pool (E_P_) using the equation FSR = [(E_2_−E_1_)∙100]/[E_p_∙time] and expressed as %/h. Muscle intracellular tissue fluid enrichment is shown in Figure S2. This calculation was completed for pre‐exercise biopsies (basal) and post‐exercise biopsies. Fold change was calculated by post‐exercise FSR/basal FSR.

### 2.5 RNA sequencing

RNA was extracted from pulverized muscle biopsy samples at baseline (pre‐exercise) and 3.5‐h after exercise (post‐exercise) using the Qiagen RNeasy mRNA extraction kit and sent to the Mayo Clinic Genome Analysis Core for sequencing as previously described (Kunz et al., 2019; Lalia et al., 2017). Briefly, libraries were prepared using the Illumina Stranded mRNA Prep and sequenced on an Illumina HiSeq 4000. Reads were aligned using STAR RNA‐seq aligner. Differential expression analysis was carried out using a negative binomial generalized log‐linear model in the edgeR R package. Gene set enrichment analysis was performed using Broads GSEA software. All gene sets that were negatively enriched with an FDR corrected *p*‐value (using the Benjamini‐Hochberg procedure) < 0.05 were reported.

### 2.6 Quantitative RT‐PCR

Quantitative real time polymerase chain reaction (qRT‐PCR) was performed on transcripts of interest that were identified in the RNA‐Seq dataset using procedures which have been previously described (Lalia et al., 2017). Total RNA was isolated using the RNEasy fibrous tissue kit according to the manufacturer's instructions. RNA quantity and purity were assessed by spectrophotometric analysis (Nanodrop) in which both the ratios of absorbance at 260 nm to that at 230 nm (A260/230) and the absorbance at 260 nm to that at 280 nm (A260/280) were >1.8. cDNA synthesis was performed using SuperScript III First‐Strand Synthesis System for RT‐PCR cDNA Synthesis Kit (Invitrogen), according to the manufacturer's protocol. The cDNA‐equivalent of 5 ng RNA was used for amplification in 384‐well microtiter plates in a QuantStudio 7 cycler (Applied Biosystems) using SYBR green assays. Cycle threshold (CT) values for individual reactions were normalized against β2 microglobulin expression (basal expression). All cDNA samples were amplified in triplicate. Post‐exercise data are presented as fold change compared with basal values using the 2‐∆∆CT method (Livak & Schmittgen, 2001). The gene‐specific primers are presented in Table 2.

**TABLE 2:** Primer sequences for quantitative RT‐PCR of muscle tissue

Gene	Primer 1	Primer 2
PGC1A	TGT CTG TAT CCA AGT CGT TCA C	GAG TCT GTA TGG AGT GAC ATC G
PGC1B	GCC TCT TTC AGT AAG CTG TCA	GCC CAG ATA CAC TGA CTA CG
PRKAA1	ACA GCT ACT TTA TGC CCA GTC	AAG ATC GGC CAC TAC ATT CTG
TFAM	GCC AAG ACA GAT GAA AAC CAC	TGG GAA GGT CTG GAG CA
HKII	TCT TAT GTA GAC GCT TGG CAA	GCA TCA AGG AGA ACA AAG GC
PDK4	CAT CTG GGC TTT TCT CAT GGA	TCC CGA CCC AAT TAG TAA ATA CC
SLC	CCC AAT GTT GTA CCC AAA CTG	TCC AAC AGA TAG GCT CCG AA
TBC	TTT CTC CCT TCT CCA TCA CTT G	TCT TCA CAC TTC CTT CTC TGC
IRS	GCA TCG TAC CAT CTA CTG ATG AG	AGT AGC TCA ACT GGA CAT CAC
MSTN	TCG TGA TTC TGT TGA GTG CT	TGT AAC CTT CCC AGG ACC A
MAPK	CTT CTT CAC TGC CAC ACG TA	GAA CAA GAC AAT CTG GGA GGT
MYOD	CCG CTT TCC TTA ACC ACA AAT C	CCG GCT GTA GAT AGC AAA GT
TRIM32	CTA CTG TCA GCC ACG ATG AG	CCA GAT TAG CCA CTT CTT CTC G
FOXO3	CTC GGC GAA GGA GAA GC	GAG GAG GAA TGT GGA AGG TG
FBX032	TCA GCC TCT GCA TGA TGT TC	CAA CAG ACT GGA CTT CTC AAC T
B2M	CCA GCG TAC TCC AAA GAT TCA	TGG ATG AAA CCC AGA CAC ATA G

Abbreviations: B2M, beta‐2‐microglobulin; FBXO32, F‐box protein 32; FOXO3; forkhead box O3; HKII, mitochondrial hexokinase II; IRS, insulin receptor substrate 1; MAPK, mitogen‐activated protein kinase; MSTN, myostatin; MYOD, myogenic differentiation 1; PDK4, pyruvate dehydrogenase kinase 4; PGC1A, peroxisome proliferator‐activated receptor gamma coactivator 1‐alpha; PGC1B, peroxisome proliferator‐activated receptor gamma coactivator 1‐beta; PRKAA1, protein kinase AMP‐activated catalytic subunit alpha A; SLC, Solute Carrier; TBC, TBC1 domain family member 1; TFAM, Transcription Factor A; TRIM 63, tripartite motif containing 32.

### 2.7 Plasma metabolomics

Amino acids and their metabolites were measured by LCMS as previously described (Lanza et al., 2010). Briefly, 20 μl of plasma was spiked with an internal standard solution consisting of isotopically labeled amino acids. The supernatant was immediately derivatized with 6‐aminoquinolyl‐N‐hydroxysuccinimidyl carbamate according to Waters' MassTrak kit. A 10‐point calibration standard curve underwent similar derivatization procedure after the addition of internal standards. Both derivatized standards and samples were analyzed on a Thermo Quantum Ultra triple quadrupole mass spectrometer coupled with a Waters Acquity liquid chromatography system. Data acquisition was done using select ion monitor (SRM) via positive electrospray condition. Concentrations of 42 analytes of each unknown were calculated against its perspective calibration curve.

### 2.8 Statistical analysis

Data were assessed for normality using the Shapiro‐Wilks Normality test. Outliers were defined by the ROUT Method with the False Discovery Rate (Q) set >1% and excluded from analysis (Motulsky & Brown, 2006). Bivariate correlation analyses were performed to examine the relationships between BMI, exercise testing, systemic inflammation, protein synthesis after exercise, and basal plasma amines. Unpaired Welch's *t*‐tests were conducted to compare outcomes for individuals with BMI < 30 kg/m^2^ (non‐obese) and individuals with BMI > 30 kg/m^2^ (obese). Two‐way ANOVAs (time and group) were conducted to compare basal and post‐exercise protein synthesis, post‐exercise plasma amines, and targeted transcriptomic data. Statistical significance was set a priori at *p* < 0.05 and analyses were performed using GraphPad Prism (v9, GraphPad Software). Data presented are mean ± SD.

## 3 RESULTS

### 3.1 Exercise test

Data from the graded exercise test (Table 3) revealed peak oxygen consumption (VO_2_peak) was similar between NO and O (*p* = 0.971) while VO_2_peak relative to total body mass was 32% lower in O compared to NO (*p* < 0.001) and negatively associated with BMI (*r* = −0.810; *p* < 0.001). VO_2_peak relative to lean body mass trended lower (*p* = 0.058) in O compared to NO and was negatively associated with BMI (*r* = −0.534; *p* < 0.001). No differences were observed in peak power, peak respiratory exchange ratio (RER), peak heart rate, and percent of age predicted maximal heart rate between NO and O. During the acute exercise bout, no differences were observed between NO and O regarding exercising workload and total work completed during the acute exercise bout (*p* = 0.160 and 0.118, respectively). Total work (*r* = −0.356, *p* = 0.026) and final rating of perceived exercise (RPE) (*r* = −0.379, *p* = 0.017) were correlated with BMI.

**TABLE 3:** Exercise data including the single leg graded exercise test and the acute exercise bout

	Non‐obese (NO)	Obese (O)	*p* value	Correlation with BMI
Graded exercise test	*N* = 19	*N* = 21		
Absolute VO_2_ peak (L/min)	1.86 ± 0.43	1.86 ± 0.44	0.971	*r* = −0.109 *p* = 0.502
Relative VO_2_ peak (ml/kg/min)	25.7 ± 4.4*	17.6 ± 4.5	<0.001	*r* = −0.810 *p* < 0.001
Relative VO_2_ peak to LBM (ml/kg LBM/min)	38.7 ± 4.8	35.0 ± 7.1	0.058	*r* = −0.534 *p* < 0.001
RER	1.16 ± 0.06	1.15 ± 0.11	0.652	*r* = −0.020 *p* = 0.901
HR_max_ (beats per min)	160 ± 16	156 ± 16	0.368	*r* = −0.251 *p* = 0.123
Age predicted HR_max_ (%)	88 ± 8	87 ± 7	0.679	*r* = −0.206 *p* = 0.326
Peak power (watts)	94.4 ± 23.3	83.7 ± 25.2	0.171	*r* = −0.301 *p* = 0.059

Acute exercise bout	*N* = 18	*N* = 21		
Acute exercise workload (Watts)	66.8 ± 16.8	59.0 ± 17.5	0.160	*r* = −0.312 *p* = 0.054
Total work during acute exercise (kJ)	119 ± 30	103 ± 32	0.118	*r* = −0.356 *p* = 0.026
Final RPE	17.2 ± 1.9	16.0 ± 2.6	0.100	*r* = −0.379 *p* = 0.017

*Note*: Data presented are mean ± SD.

Abbreviations: LBM, Lean Body Mass; RER, Respiratory Exchange Ratio; RPE, Rating of Perceived Exertion.

* *p* < 0.05 versus O.

### 3.2 Baseline and post‐exercise muscle protein synthesis

**FIGURE 2:**
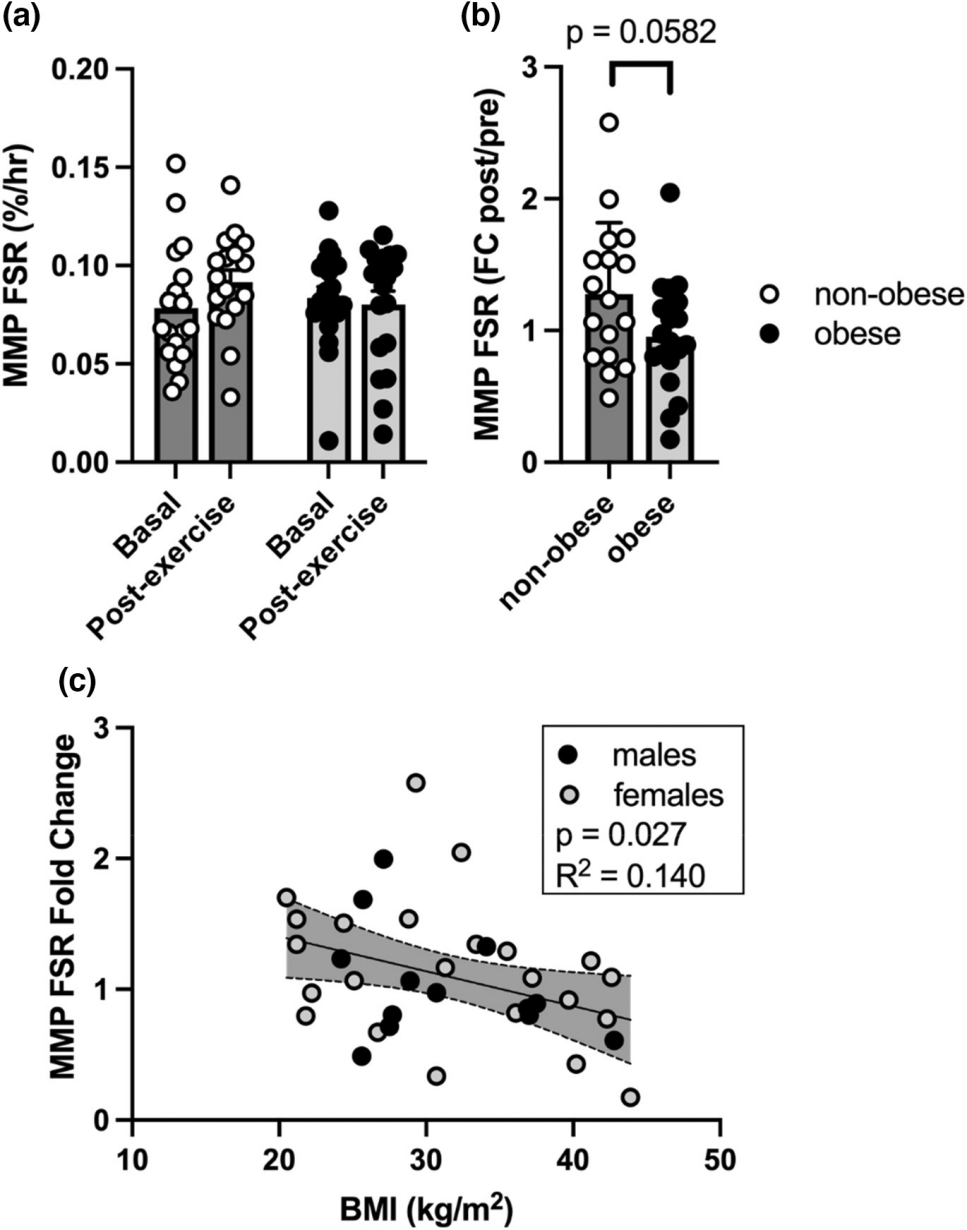
Basal and exercise‐induced muscle protein synthesis. Mixed muscle protein (MMP) fractional synthesis rate (FSR) was measured from the rate of incorporation of isotopically labeled ^13^C_6_ phenylalanine into muscle proteins in non‐obese adults (NO; *N* = 17) and adults with obesity (O; *N* = 19). FSR was measured at baseline (postabsorptive) and 3.5 to 6.5 h following a single bout of aerobic exercise (a). No differences were observed between NO and O in MMP basal (*p* = 0.164) and post‐exercise FSR (*p* = 0.230). Exercise‐induced fold change (FC) of MMP FSR trended (*p* = 0.0582) between NO and O (b). To further interrogate this trend, regression analysis revealed a significant negative relationship between exercise induced MMP FSR and BMI, regardless of sex (c).

Basal postabsorptive muscle protein fractional synthesis rates (FSR) were similar (*p* = 0.164) in people with and without obesity (Figure 2a). The anabolic response to exercise in skeletal muscle was determined from the fold change in FSR from rest to post‐exercise. A statistical trend (*p* = 0.058) for lower FSR fold‐change was observed in people with obesity (0.955 ± 0.423) compared to people without obesity (1.276 ± 0.542) (Figure 2b). The induction of muscle protein FSR after exercise was negatively associated with BMI (*p* = 0.027, *r* = −0.375) with no differences between males and females (Figure 2C).

### 3.3 Skeletal muscle transcriptional response to exercise

**FIGURE 3:**
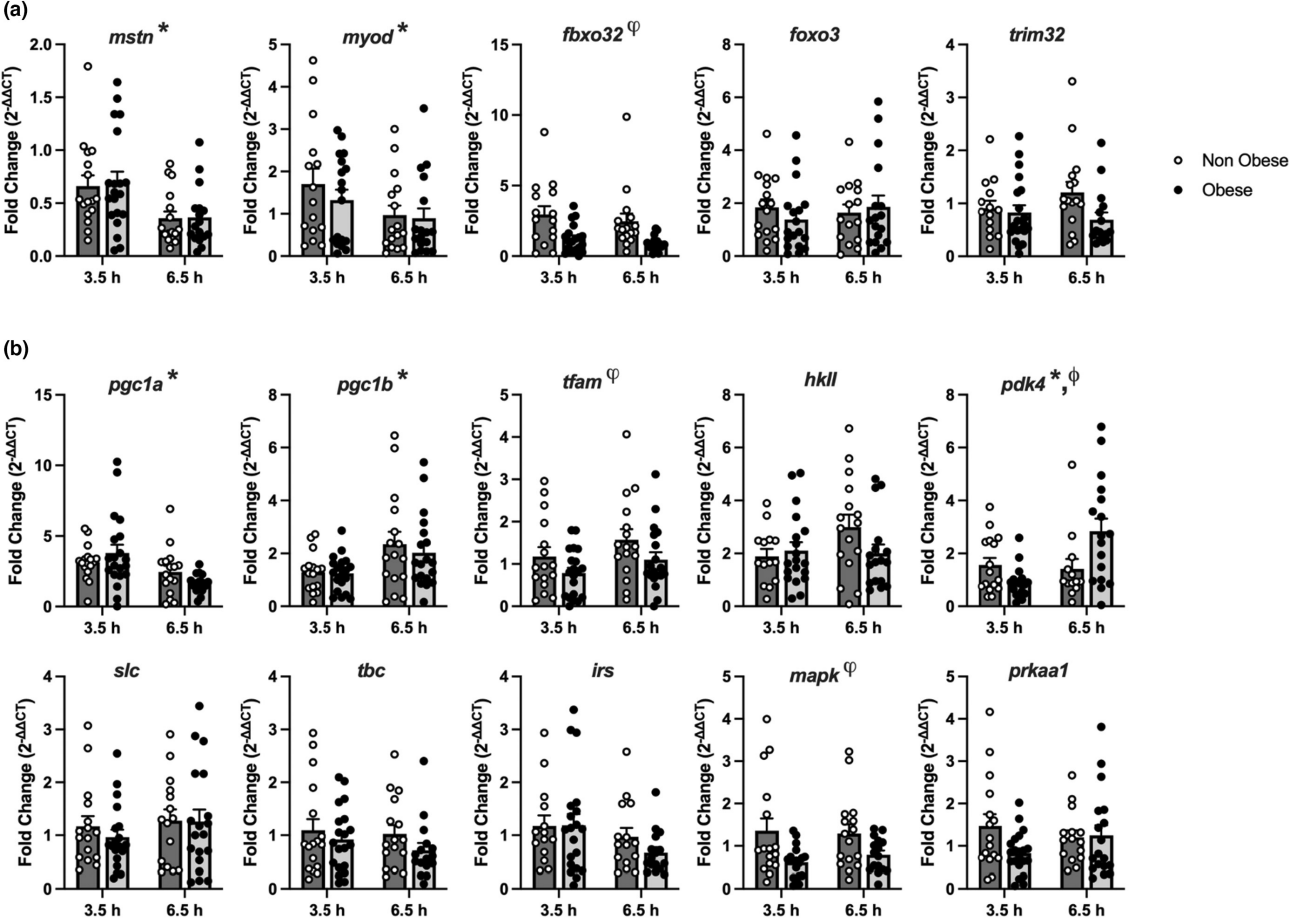
Effect of an acute bout of aerobic exercise on selected gene transcripts related to protein turnover (a) and metabolism (b). Skeletal muscle gene expression was measured in non‐obese adults (NO; *n* = 17) and adults with obesity (O; *n* = 20) by qRT‐PCR and expressed relative to their basal values (Figure S1). To quantify their response to an acute bout of aerobic exercise at different time points, we used the 2−^ΔΔCT^ method at 3.5‐ and 6.5‐h post exercise. A two‐way ANOVA was performed to compare the main effects (time and group). NO had greater responses in FBXO32, TFAM, and MAPK, while PDK4 exhibited a time and group interaction as O had a greater response at the 6.5‐h mark compared to NO. MSTN, myostatin; MYOD, myogenic differentiation 1; FBXO32, and F‐box protein 32; FOXO3; forkhead box O3; TRIM 63, tripartite motif containing 32; PGC1A, peroxisome proliferator‐activated receptor gamma coactivator 1‐alpha; PGC1B, peroxisome proliferator‐activated receptor gamma coactivator 1‐beta; TFAM, transcription factor a; HKII, mitochondrial hexokinase II; PDK4, pyruvate dehydrogenase kinase 4; PRKAA1, protein kinase AMP‐activated catalytic subunit alpha a; MAPK, mitogen‐activated protein kinase; SLC, solute carrier; TBC, TBC1 domain family member 1; IRS, insulin receptor substrate 1. ^ψ^ *p* < 0.05 NO vs O. *p < 0.05 3.5 h versus 6.5 h. ^ϕ^ *p* < 0.05 time and group interaction. Data bars are mean ± SD.

To interrogate the molecular pathways that may underly attenuated anabolic response in people with obesity, we used a combination of targeted (qRT‐PCR) and non‐targeted (RNA sequencing) analytical approaches to examine unique and common transcriptional patterns in the two groups. Using qRT‐PCR, we specifically targeted genes involved in muscle protein turnover (*mstn*, *myod*, *trim32*, *foxo3*, and *fbx032*) and energy metabolism (*pgc1a*, *pgc1b*, *tfam*, *pdk4*, *hkII*, *irs*, *mapk*, *prkaa1*, *slc*, and *tbc*) (Figure 3). The expression of these genes was measured in skeletal muscle samples collected at rest and two timepoints following acute exercise (3.5 h, 6.5 h). Of these transcripts, baseline expression of hexokinase II (*hkII*) was positively associated with BMI, whereas myostatin (*mstn*) was negatively associated with BMI (Figure S1). Distinct exercise responses were observed in people with and without obesity. Notably, the exercise‐induced expression of *mapk*, *tfam*, and *fbxo32* was attenuated in people with obesity, while *pdk4* increased in the obese cohort 6.5 h after exercise (Figure 3).

**FIGURE 4:**
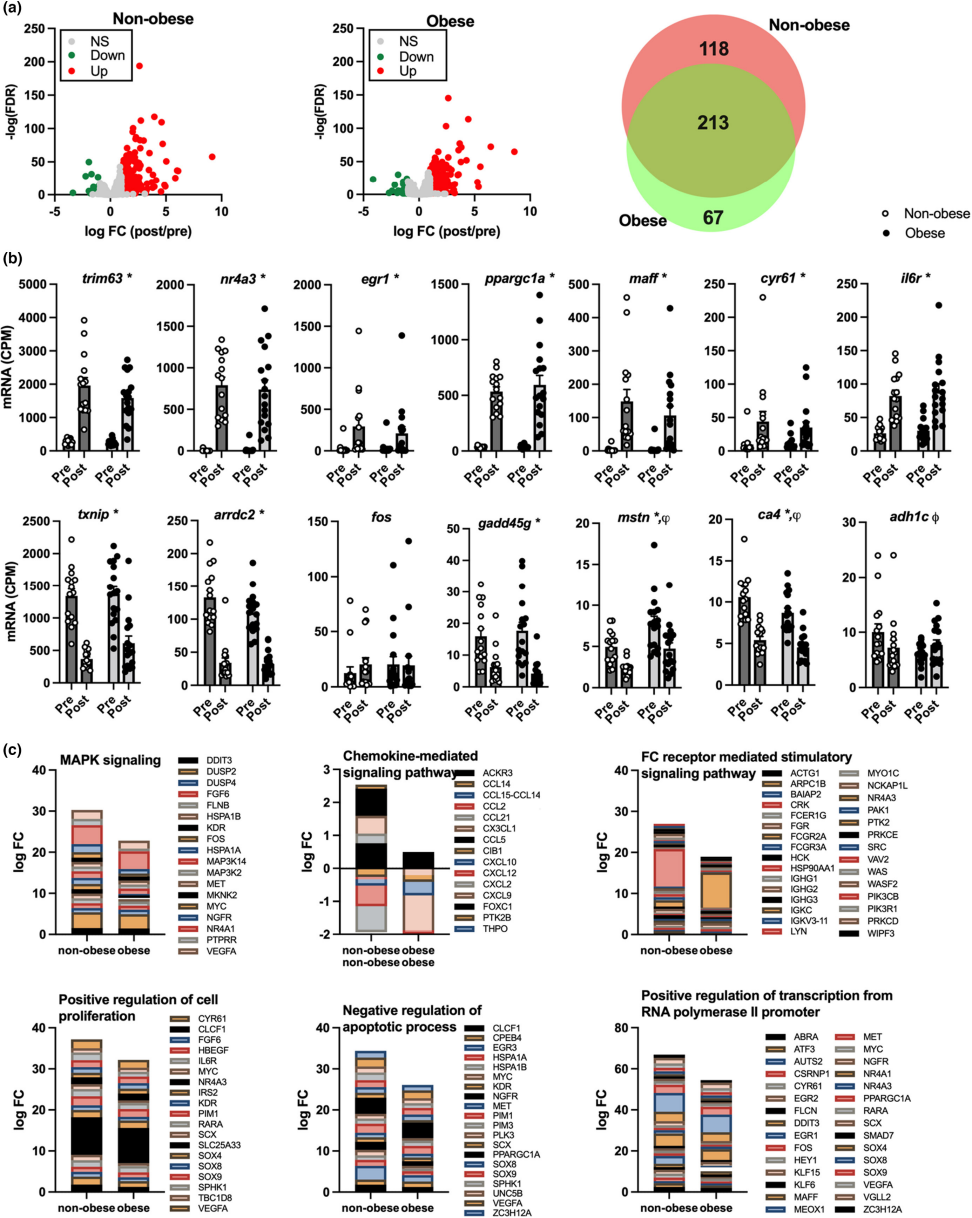
Exercise‐induced transcriptional patterns in skeletal muscle in obese and non‐obese adults. RNAsequencing was performed in muscle tissue samples collected before and after exercise in non‐obese adults (NO; *N* = 15) and adults with obesity (O; *N* = 17). Volcano plots (a) show log fold change (FC) vs. ‐log discovery rate (FDR) for transcripts that were significantly (*p* < 0.05) different between non‐obese and obese after exercise. The Venn diagram shows the number of transcripts that were similarly and differentially expressed with exercise in non‐obese and obese after exercise. We then compared gene expression between NO and O of 14 genes which have been previously shown to be responsive to acute aerobic exercise(Pillon et al., 2020) (b). Gene set enrichment analysis (GSEA) was used as an agnostic approach to determine if there were coordinated gene expression changes that differed between obese and non‐obese individuals. There were six dominant gene sets that emerged as being enriched in response to exercise in non‐obese and attenuated in obesity (c). ^ψ^ *p* < 0.05 NO vs O. *p < 0.05 3.5 hrs vs 6.5 hrs. ^ϕ^ *p* < 0.05 time and group interaction.

In addition to targeted gene expression measurements, RNA sequencing was performed at baseline and 3.5‐h after exercise to examine whole muscle transcriptional patterns and which molecular pathways may be uniquely influenced by exercise in people with and without obesity. A total of 331 genes were differentially expressed in response to exercise (36 downregulated, 295 upregulated) in non‐obese individuals while a total of 280 genes were differentially expressed in response to exercise (45 downregulated, 235 upregulated) in people with obesity (Figure 4a). Based on current knowledge base(Pillon et al., 2020), we focused on 14 candidate genes known to be responsive to acute aerobic exercise. Of these candidate genes, 7 were significantly upregulated following exercise (*trim63*, *nr4a3*, *egr1*, *ppargc1a*, *maff*, *cyr61*, and *il6r*) but did not differ between obese and non‐obese at rest or following exercise (Figure 4b). Five of the exercise responsive genes were significantly downregulated following exercise (*txnip*, *arrdc2*, *gadd45g*, *mstn*, *ca4*). Notably, myostatin (*mstn*) expression was lower in non‐obese compared to obese and adh1c expression decreased following exercise in non‐obese but increased following exercise in obese. Gene set enrichment analysis (GSEA) identified six gene sets that were distinctly influenced by acute exercise in people with and without obesity (Figure 4c). Pathways included MAPK signaling, chemokine‐mediated signaling pathway, FC receptor‐mediated stimulatory signaling, positive regulation of cell proliferation, negative regulation of apoptotic process, and positive regulation of transcription from RNA polymerase II promoter.

### 3.4 Circulating amino metabolites

**FIGURE 5:**
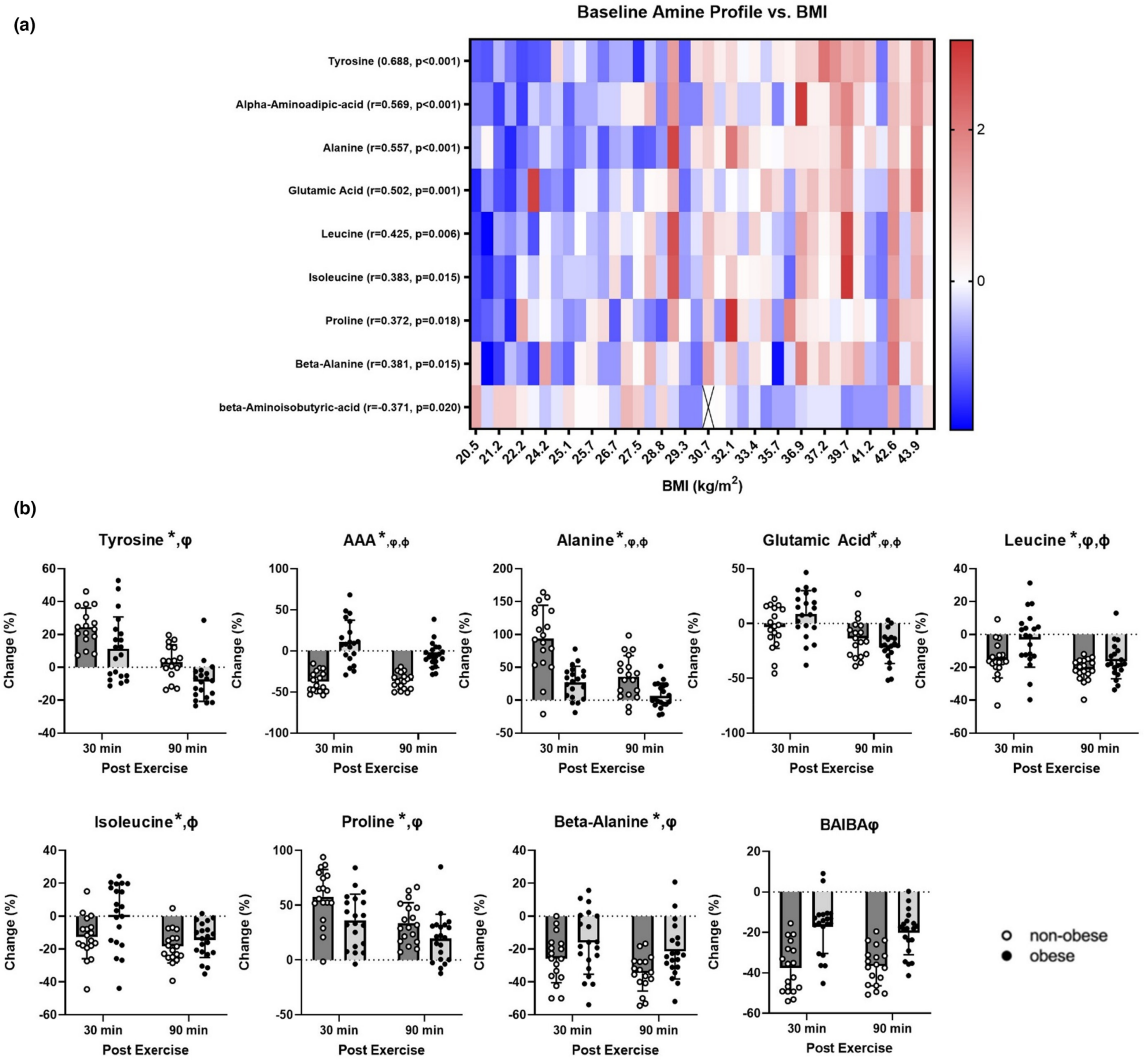
*Effects of obesity on basal and post‐exercise circulating amine metabolomics*. A total of 37 amino metabolites were quantitatively measured using a targeted mass spectrometry method in plasma at rest, 30 min after exercise, and 90 min after exercise. At the basal state, Z scores for each subject (*N* = 40) were calculated and correlated with BMI for each metabolite. Z scores of 17 amine metabolites were significantly correlated with BMI. Circulating amines were then made relative to their baseline values at 30 and 90 min after exercise to calculate percent change [(post‐pre)/post] in non‐obese adults (NO; *N* = 18) and adults with obesity (O; *N* = 20). Percent change was compared using a two‐way ANOVA (time x group) between non‐obese (NO) and obese (O) adults. 14 of the 37 measured circulating amines were influenced by obesity (effect of group and/or main effect interaction). Together, 9 amine metabolites appear to be influenced by obesity both at rest (a) and 30‐ and 90‐min post‐exercise (b). The basal correlations and post‐exercise data of all amines are shown in Table S1. ^ψ^ *p* < 0.05 NO vs O. *p < 0.05 3.5 hrs vs 6.5 hrs. ^ϕ^ *p* < 0.05 time and group interaction. Data bars are mean ± SD. Values that were identified as outliers as previously mentioned were removed and are represented with an X (*N* = 1).

We interrogated plasma amino metabolites to provide an additional window into the influence of obesity on acute exercise response molecules related to whole‐body protein and amino acid metabolism. A total of 37 amino metabolites were quantitatively measured using a targeted mass spectrometry method in plasma at rest, 30 min after exercise, and 90 min after exercise. Basal plasma amine metabolomic profiling was performed for each subject to assess. Z scores for each subject were calculated and correlated with BMI for each metabolite. At rest, 17 amine metabolites were significantly correlated with BMI (Table S1). Following exercise, 14 amine metabolites demonstrated distinct exercise response patterns in individuals with and without obesity (Table S1). In total, there were 9 overlapping amine metabolites that were influenced by obesity both at rest (Figure 5a) and after exercise (Figure 5b). These metabolites include the amino acids tyrosine, alanine, leucine, isoleucine, proline, and glutamic acid, and the amino metabolites alpha‐aminoadipic acid (AAA), beta‐alanine, and beta‐aminoisobutyric acid (BAIBA).

## 4 DISCUSSION

Physical activity is a modifiable lifestyle factor and attractive strategy to lessen the burden of obesity. Nevertheless, it is critical to understand the factors that may attenuate exercise response in the quest to optimize, individualize, and tailor adjunctive therapies to be used alongside exercise. In the context of obesity, systemic inflammation and metabolic dysregulation have been suggested to interfere with exercise responsiveness (Greiwe et al., 2001; Lessard et al., 2013; Rieu et al., 2009; Stephens & Sparks, 2015). In an effort to further characterize the influence of obesity on the exercise response, we evaluated skeletal muscle and systemic molecular responses to a single bout of aerobic exercise in people with and without obesity. The main findings of this study are: (1) the anabolic response to acute exercise is diminished in people with obesity, (2) several salient exercise‐responsive genes and pathways are attenuated following exercise in people with obesity, including metabolic regulation, cell‐to‐cell communication, and protein turnover, and (3) people with obesity exhibit unique circulating amino metabolite profiles at rest and following acute exercise.

### 4.1 Obesity influences the anabolic response to acute exercise

Muscle mass is critical for adequate physical function and good metabolic health and is a determinant of relative risk of mortality (Wolfe, 2006). While muscle hypertrophy and protein anabolism are typically associated with resistance exercise, aerobic exercise also activates anabolic response pathways in skeletal muscle in support of metabolic and structural adaptations over time (Harber et al., 2010; Miller et al., 2005; Wilkinson et al., 2008). In the current study, we show that non‐obese individuals increased muscle protein synthesis to a greater extent (~1.3‐fold) than people with obesity (~0.9‐fold) after an acute bout of aerobic exercise. Although this group difference in anabolic response to exercise did not reach statistical significance (*p* = 0.058), there was a significant negative association between BMI and the fold‐induction of muscle protein synthesis following exercise, regardless of sex. The magnitude of fold‐induction of muscle protein synthesis observed with single leg cycling in the current study is consistent with previously published muscle protein synthesis following 60 min of cycling exercise in young, active men (Harber et al., 2010). In contrast, Serrano et al. reported no change in muscle protein synthesis in adults with or without obesity after 45‐min of cycling (Serrano et al., 2021). Key considerations when comparing the magnitude of anabolic response across studies include the time course of muscle sampling, subject characteristics, and exercise intensity/duration. It is important to note that post‐exercise anabolic response was not associated with exercise workload in the current study (*p* = 0.977, *r* = 0.006), suggesting that the diminished exercise response with increasing BMI cannot be explained by differences in exercise performance. To date, the influence of obesity on skeletal muscle anabolic response has been mostly studied from the standpoint of nutritional stimuli (Beals et al., 2016, 2019; Guillet et al., 2009; Paulussen et al., 2021), but the effects of obesity on the anabolic response to exercise are less clear. A prior study by Beals and colleagues demonstrated blunted muscle protein synthesis and anabolic signaling pathways in response to resistance exercise and feeding in people with obesity (Beals et al., 2018). Although the current study measured post‐exercise muscle protein synthesis under postabsorptive conditions, the results are consistent with the earlier study by Beals et al. that included a feeding response after exercise, and in agreement with the notion that anabolic response to acute exercise is attenuated in people with obesity. The mechanisms contributing to anabolic resistance with obesity continue to be interrogated, although there is good evidence for altered signaling through p70S6K (Beals et al., 2018), which may be consequent to heightened levels of inflammatory molecules in circulation or in muscle tissue. The rationale for this hypothesis is based on our observations that muscle anabolic response was negatively associated with plasma TNFa levels (*p* = 0.004, *r* = −0.467); an observation that has been reported previously in aging (Toth et al., 2005).

### 4.2 Exercise‐induced transcriptome response is different in adults with and without obesity

To further interrogate the exercise response in adults with obesity, we conducted both non‐targeted (RNA sequencing) and targeted (qRT‐PCR) analysis on mRNA expression of the vastus lateralis after exercise. Whole‐muscle transcriptomics revealed that the majority of the transcripts differentially expressed in response to exercise were common in people with and without obesity (213 transcripts), but an additional 118 transcripts were uniquely influenced by exercise in non‐obese and 67 uniquely influenced in people with obesity. As a first step, we examined a small number of canonical exercise‐response transcripts, including the top 5 up‐ and down‐regulated genes that were recently curated from 12 published datasets from acute aerobic exercise studies in humans (Pillon et al., 2020). This curated set of genes demonstrated the expected increase or decrease in expression based on published literature; an observation that reinforces that the current single‐leg exercise protocol was sufficiently robust to recapitulate known gene expression changes in skeletal muscle. Of these genes, myostatin (*mstn*) and carbonic anhydrase IV (*ca4*) were differentially expressed in people with and without obesity, but only alcohol dehydrogenase 1c (*adh1c*) demonstrated a significant interaction whereby expression decreased in response to exercise in people without obesity but increased in people with obesity. It is currently unclear how this gene may be involved in distinct acute molecular responses to exercise in people with or without obesity. We used qRT‐PCR to specifically target a number of transcripts known to regulate muscle protein turnover in response to exercise. Of these, only F‐Box protein 32 (*fbxo32*) demonstrated attenuated expression following exercise in people with obesity. Also known as Atrogin‐1, this gene encodes a protein that is a subunit of a ubiquitin protein ligase that is involved in muscle protein breakdown and atrophy (Bodine et al., 2001). The upstream transcription factor *foxo3* did not demonstrate any obesity‐related changes in exercise response, nor did the ubiquitin ligase *trim32*. Whether the distinct pattern of fbxo32 expression following exercise has any relevance to the observed differences in muscle anabolic response to exercise in people with or without obesity remains to be determined.

Pyruvate dehydrogenase kinase 4 (*pdk4*) was notable as an exercise‐responsive transcript that demonstrated distinct expression patterns in people with and without obesity. PDK4 influences muscle substrate utilization through its deactivation of pyruvate dehydrogenase, leading to a shift away from carbohydrate oxidation in favor of fat oxidation. PDK4 has been previously shown to increase acutely after exercise (Pilegaard et al., 2005), but the observed pattern whereby people with obesity exhibited a heightened but delayed post‐exercise increase in *pdk4* gene expression suggests that late post‐exercise substrate metabolism is shifted toward lipid metabolism in people with obesity. This may suggest the obese group had poor glucose disposal within the muscle or elevated circulating fatty acids in recovery compared to the non‐obese group, overall suggestive of poor metabolic recovery after exercise. While others reported the regulation of glycogen synthesis is preserved after exercise in insulin‐resistant subjects (Jensen et al., 2012), these data, along with our previously published work on dysregulated glycogen storage after exercise in adults with obesity (Vanderboom et al., 2022), suggests more research is warranted.

Mitogen‐activated protein kinase (*mapk*) was induced in skeletal muscle following exercise, but to a lesser extent in people with obesity. Furthermore, gene set enrichment analysis identified MAPK signaling as a pathway that was strongly influenced by exercise but attenuated in people with obesity. The MAPK pathway is a key component in exercise‐induced metabolic adaptations (Raney & Turcotte, 2006; Widegren et al., 1998; Yu et al., 2003) and has been shown to be upregulated in aerobic exercise in humans (Boppart et al., 2000; Widegren et al., 1998; Yu et al., 2001). Given this pathway appears to be activated by exercise in response to oxidative, energetic, and/or mechanical stress (Kramer & Goodyear, 2007), there appears to be a different oxidative and/or metabolic stress within the muscle during exercise between adults with and without obesity and is a prime target for future investigations. Chemokine mediated signaling, is another relevant pathway as myokine secretion is related to metabolic crosstalk with other tissues (Laurens et al., 2020), and has large implications for health and metabolic diseases (So et al., 2014). While these two pathways are likely related to metabolic adaptations, the others are related to immune response and protein turnover and may be related to the basal systemic inflammation in the adults with obesity.

We previously reported dysregulated post‐exercise GSK3β signaling in adults with obesity which may suggest lower basal muscle glycogen content (Kunz et al., 2021; Standl et al., 1980; Vanderboom et al., 2022). Additionally, during exercise, Goodpaster et al. reported that men with obesity exhibited lower rates of muscle glycogen oxidation and higher fatty‐acid oxidation from non‐plasma sources compared to men without obesity (Goodpaster et al., 2002). Although glycogen levels were not measured in the present study, it is difficult to ignore the possibility that metabolic dysregulations prior to, during, and/or after exercise contribute to varying exercise response in people with obesity. It is likely that these metabolic dysregulations and/or the basal inflammatory profile contribute to the blunted exercise response seen in these adults with obesity and warrant more mechanistic investigation.

### 4.3 Circulating amine metabolomics reveal different basal and post‐exercise substrate availability

We measured circulating plasma amino metabolites before and 30‐ and 90‐min post exercise to evaluate systemic metabolite markers of exercise response. We found 17 circulating amines that were significantly associated with BMI at rest and 14 that were influenced by exercise in an obesity‐dependent manner. Notable metabolites that were influenced by obesity and exercise were tyrosine, alanine, leucine, isoleucine, proline, glutamic acid, alpha‐aminoadipic acid (AAA), beta‐alanine, and beta‐aminoisobutyric acid (BAIBA). Previous studies have shown adults without obesity had lower circulating total and essential amino acids 3 h after aerobic exercise while adults with obesity had no change from baseline (Serrano et al., 2021). Of the essential amino acids, we report adults with obesity had blunted post‐exercise declines in isoleucine and leucine compared to people without obesity. Leucine is noteworthy for its role in protein synthesis and modulator of the insulin phosphoinositide 3‐kinase signal cascade, a key pathway in insulin signaling (Norton & Layman, 2006). In a prior report, circulating leucine decreased by ~13% 30‐min post exercise (Makhro et al., 2016), which is consistent with what was observed in adults without obesity in the current study. Circulating alanine and glutamic acid are indicators of glycolytic flux (Henriksson, 1991) and known to increase in plasma following exercise (Bergstrom et al., 1985; Einspahr & Tharp, 1989; Makhro et al., 2016). Post‐exercise alanine levels did not rise as much in people with obesity compared to people without obesity, which may be secondary to dysregulated glucose metabolism following exercise in the obese group. In support of this hypothesis, Blomstrand & Saltin reported a greater release of alanine in exercising muscle with normal glycogen content compared to exercising muscle with low glycogen content (Blomstrand & Saltin, 1999). Notably, no significant differences were observed after exercise in phenylalanine or 3‐methylhistidine, which along with tyrosine, are common markers of muscle protein breakdown (Henriksson, 1991).

In addition to amines related to protein turnover and metabolism, several other amines were significantly associated with BMI and altered in response to exercise. Importantly, some of these metabolites have been shown to be prognostic indicators for diseases. Tyrosine, leucine, and cysteine were three amines with robust associations with BMI (*r* = 0.688, *r* = 0.425, and *r* = 0.433, respectively), and have been previously suggested as early biomarkers for metabolic syndrome (Lynch & Adams, 2014; Mohorko et al., 2015). BAIBA is a catabolite of thymine and valine metabolism that has been shown to be inversely associated with cardiometabolic risk, released from muscle in response to exercise and PGC‐1alpha overexpression, induces adipose browning, and improves glucose metabolism (Roberts et al., 2014). Others report that circulating BAIBA levels increase in humans with exercise training (Roberts et al., 2014) and acutely following a single bout of exercise (Stautemas et al., 2019). Consistent with precedent literature, we find that plasma BAIBA levels decrease with increasing BMI, however, plasma BAIBA levels were acutely lower 30 min and 90 min after exercise. We did not measure BAIBA during or immediately post‐exercise, which is important because this metabolite appears to rapidly return to baseline values within 30 min (Stautemas et al., 2019). Alpha‐aminoadipic acid (AAA) is another interesting metabolite of lysine that is predictive of diabetes risk (Wang et al., 2013). We also find that AAA is positively associated with BMI and is acutely decreased following exercise in non‐obese, but not in people with obesity. The link between AAA and metabolic disease, combined with our new observations that AAA is responsive to acute exercise in an obesity‐dependent manner suggests that this is a metabolic pathway that is particularly dysregulated with obesity and may be worth follow‐up investigations to better understand the biological relevance and whether it is worth targeting this pathway in people with obesity.

### 4.4 Limitations and future directions

In our quest to characterize salient exercise response parameters in people with obesity, we acknowledge some limitations to this study and suggest areas for future research. First, we measured protein synthesis between 3.5 and 6.5 h following exercise in an effort to capture peak anabolic response based on prior timecourse studies (Fry et al., 2011; MacDougall et al., 1995). It is possible that the peak anabolic response to exercise fell outside this window in this study, in which case additional biopsy timepoints would be required to more confidently identify the time at which peak muscle protein synthesis occurred. Another important consideration is that anabolic response to exercise was determined from measurements of mixed muscle protein synthesis, which reflects a large number of individual proteins that may respond differently to exercise. Inasmuch, we acknowledge that additional measurements of subcellular protein pools or even synthesis rates of individual proteins would provide additional novel insights into the fundamental influences of obesity on muscle protein synthesis. Lastly, we used a single‐leg exercise protocol in an effort to minimize complications related to exercise following serial muscle biopsies. While conventional bilateral cycling is more practically relevant, we would not expect muscle‐specific outcomes between the groups to be impacted. However, it is likely that conventional bilateral cycling would exert a more striking influence on the plasma metabolomic signatures and perhaps reveal additional distinctions in exercise response between people with and without obesity.

## 5 CONCLUSION

In conclusion, these data demonstrate that people with obesity exhibit modestly attenuated exercise‐induced muscle protein synthesis in conjunction with notable shifts in exercise‐responsive gene transcripts, including muscle protein breakdown, substrate metabolism and fuel selection, and oxidative/metabolic stress response pathways. The heterogeneity of exercise responsiveness in humans and the existence of “exercise resistant” populations necessitate new ways to individualize exercise prescriptions or develop adjunctive strategies to help enhance exercise response by targeting pathways that attenuate molecular exercise response pathways in people with obesity.

## AUTHOR CONTRIBUTIONS

Conceived and Designed Research: I.R.L. Performed Experiments: K.J.G., C.R.H., H.E.K., Z.R., X.Z., I.R.L. Analyzed Data: K.J.G., H.E.K., Y.L, S.D., I.R.L. Interpreted Results of Experiments: K.J.G and I.R.L. Prepared Figures and Drafted Manuscript: K.J.G. and I.R.L. Edited and Revised Manuscript: K.J.G., C.R.H., H.E.K., Z.R., X.Z., Y.L, S.D., I.R.L. Approved Final Version of Manuscript: K.J.G., C.R.H., H.E.K., Z.R., X.Z., Y.L., S.D., I.R.L.

## ETHICS STATEMENT

This study was approved by the Institutional Review Board at Mayo Clinic (IRB# 16‐000437). All subjects provided written informed consent.

## FUNDING INFORMATION

This project was supported by Grant Number UL1 TR002377 from the National Center for Advancing Translational Sciences (NCATS). Its contents are solely the responsibility of the authors and do not necessarily represent the official views of the NIH.

## CONFLICT OF INTEREST

The authors declare they have no competing interests.

## Supporting information


Figure S1
Figure S2
