# Supplementary material for: Acute responsiveness to single leg cycling in adults with obesity
Source: Physiol Rep. 2022 Dec 21;10(24):e15539. doi: 10.14814/phy2.15539 (PMC9768637; doi:10.14814/phy2.15539)
Supplement: Supplementary file 1 — Figure S1 Figure S2 [file PHY2-10-e15539-s001.docx]

**Supplemental Figure 1**

**Supplemental Figure 2**
